# Workday Playdays

**Published:** 1874-04

**Authors:** Ben. H. Pratt


### THE BISTOURY.

Vol. X. ELMIRA, N. Y., APRIL, 1874. No. 1.

### Contributions.

### WORKDAY PLAYDAYS.

BEN. H. PRATT, A. M.

Our theories, like our books, by and by become dog-eared and grimy. We eagerly and proudly displayed their newness once—were as vain of our new hobby as a school boy of his first primer—opened its pages carefully—examined into the cleanliness of our hands ere we took it up—sat to its perusal with an avidity that betrayed our actual love for it—were jealous of our possession and pitied our unfortunate mate who owned no such boon. In use it was covered carefully, and the mark and the thumb paper were ever in place. When duty called us from it, we regretfully gave it up for the time being, our thoughts dwelling upon the early return of the sweet privilege of renewing our communings with it. Then we laid it carefully by, out of harm's way, and beyond the reach of vandal hands represented by the several pairs of juvenile fists, lest perchance our prize become like unto the leafless and oleaginous specimens that served our schoolmates the double purpose of study and defense.

But the newest hobby—the freshest theory—the latest idea—like the book, will soil at the edge—curl up at the corners—lose its newness and its freshness, and finally go the way of all hobbies, all theories, all pet ideas, and after suffering the ills of our indifference, lapse into utter disregard and by and by fall beneath contempt.

The man, like the boy, must have its new plaything. Unlike the boy, however, his play must get him bread, and that the bread getting become not irksome, he is continually revolving within his own mind how he can vary his toy without diminishing his modicum of daily food. This endeavor to convert what we are accustomed to call work, into what we may feel to be play, is the great problem of life to those who must get, somehow, somewhere and somewhence the meat that perisheth, but, in perishing, sustains. He whose work is all work, is a slave. He whose work is all play, is the only happy man. Men are graduated between the extremes of happiness and misery, and stand above or below the mean or middle point, in proportion to the excess of their play or their work. Hence we find that the best brain power is expended selfishly, though it may result in wondrous benefits to our kind. He who has the faculty of adapting that peculiar thing we call "business," "occupation," "employment," so nicely to his temperament that it is not irksome, but a continual source of pleasure to him who pursues it, that man has attained to about the highest type of happiness, other things being equal, vouchsafed to mortals. He has indeed found the true philosopher's stone, which, though it may not convert what it touches into gold, does more than this, since it converts life into joys that gold itself cannot purchase.

The narrow thoughted, then, is he who has made up his mind definitely that work is one thing and play its opposite—who devotes so much of life as he can afford to what he denominates work, and relies upon what play he can catch on the wing, for his relaxation, his recreation, his recuperation. He thinks himself a philosopher, forsooth, because he has discovered that he can work a certain number of hours, sleep a certain number of hours, and play a certain number of hours, and retain his health, make a living, and have the average amount of pleasure allotted to men, between times. He admits that his work is irksome, as he claims all work is—that sleep is necessary—that play is a counter agent to neutralize the effects of work. He deems it a special and exceedingly wise provision of Providence that play has been instituted, for thereby work is rendered tolerable. His error is in the characteristics he gives to the thing he calls work. He endows it with no attractions —he weaves no fascinations about it—he sees in it the inevitable only. He does not shrink from it—does not dread nor fear it—enters upon its duties and requirements with a perfect reconciliation—accepts the necessity without murmur, believing that it is the result of the curse pronounced upon Adam, to whom it was ordained that "by the sweat of thy brow thou shalt earn thy bread." He is convinced that sweat and happiness cannot be convertible terms, and that all labor, all work, all effort of whatsoever kind it be, is a thing to suffer patiently and be endured murmurless. He don't complain of his lot, perhaps, nor deem himself less fortunate than his fellows, but he entertains a very narrow and, we claim, a very mistaken idea of the place work holds in the grand economy of nature and in the sublime diapason of humanity.

We grant that owing to the varied circumstances of life, men are often compelled to do that which is absolutely repugnant to their tastes and natures. Man is not always the abiter of his own relations to the varied and complicated interests into which circumstances may have cast him. He may be compelled to pursue a line of occupation that is not consonant with his tastes, and that comes into daily antagonism with his own convictions of his occupational requirements, and yet be unable to discover just the place into which his nature surely fits. We go so far in our theory of business adaptation as to believe that every man born into the world was intended for, and fitted to, a special place in the mundane economy—a place, in which if he were to get, the world would gain by his position, and he would fill that place with the same enjoyment that he feels when he takes part in what he now terms play and recreation —a place in which work is play, that is, deprived of everything that can make it in the least irksome. We have a conviction, founded upon analogy in nature, that no man was ever created for a life of idleness, be his station however high it may; that healthful humanity cannot contentedly be idle; that work will be sought for even by him whose needs are superior to the necessity therefor; that it is as necessary to completeness of living as is the food he eats; that in some form or other, that which we call work enters as naturally into the life element, as muscular and mental exercise enter into the health element; that life and health demand work as the grand essentials of their well being.

But that which is work to one man is play to another, and *vice versa.* The discontented man, whose brow reeks with the sweat of hated toil, does not envy the position of the astronomer whose nightly gaze is on the firmament in search of a planet whose presence there his calculations as surely tell as though Deity had whispered it in his ear. Both of these are workers, but while the former curses his own lot and envies the gay butterfly of society, whose shallow pate sees naught on earth attractive save the flittings from one social flower to another, he grieves for the poor old mathematician, the happiest man on earth, perhaps—happy because his work is play, and a play around which more fascinations linger than ever waited upon the possession of infinite leisure supported by regal wealth. The play of the boy is work and his work is play. No happier being exists than the boy who strives to imitate his elders' work. He 'll tug and strain at "playing man," and do work that a man would shrink from, calling it the jolliest fun agoing. Wonders of work have been accomplished by knowing men inveigling boys into the idea that it was play. Set a boy at clearing brush and picking stones from new land, and he whimpers and goes at it like a slave driven under the fear of the lash. But call about you half a dozen boys and suggest what fun 'twill be to build a huge chimney of the stones lying about, and then burning therein all the brush and logs and old stumps upon the field, and you 'll get a week's hard work out of those boys' play.

He whose work, then, is not attractive to himself—has no charms beyond the remuneration he gets for it—goes to it laggingly and accomplishes it per force of will only—wishes he were rich and didn't have to work—sees no good in, and gets no good from, his occupation, save a consciousness of time well spent, a duty passably performed, a conventional claim complied with—such an one is not in his place. By this consciousness shall he know it, and from these warnings let him carefully consider his qualifications and tastes and seek such work as shall not be irksome—such work as he would rather engage in and prosecute, than hold the most independent position on earth.

The man who tells you how differently he will live when he gets rich—how quickly he will lay aside all the insignia and mementoes of his present mode of life so soon as he shall be able so to do—this man is out of his place.

He only is in his proper and appropriate sphere who would not abandon his business, however high a position he might attain among the men of wealth—however unnecessary it might be for him to raise a finger for his future needs—who would keep right on in his present career, enjoying his work the better, the less necessary it became for him to engage in it—whom no tawdry attractions can draw from his favorite occupation—whom no one can coax to abandon it. This devotion to work, be it what it may, a trade, a profession, an art, a science or what not, is one of the facts that stump men who have mistaken their calling. Such sneer at the rich merchant because he persists in doing business at three score and ten, with his millions already invested in fabulously paying stocks. They quote in derision the strict exactions of the affluent banker, whose business habits the growth of a life of business had compelled to do business in a business way. They charge the opulent manufacturer with base stinginess because he exacts but justice from the operative, and do not appreciate the bond that binds him to his life work. Men cannot conceive why the man wedded to an art would rather live on crusts the balance of his days than accept the proudest position in the gift of the people at the sacrifice of his loved pursuit.

Onerous and repellant as most of us may deem many occupations, there is not, in the whole catalogue of work, anything so mean in our eyes, so filthy, so difficult, so intricate, so trying, so dangerous, so confining, that there are not dipositions and temperaments and tastes adapted thereto. The deftly gifted manipulator of the harp or piano looks aghast upon the bared and brawny iron worker, and is almost pitied, in return, for his effeminacy. The dapper and sleek financier holds his nose and gives a wide berth to the passing scavenger and swill peddler, who enjoy as heartily their highly flavored investigations as he enjoys a contemplation of his bank book. They would not be he if they could, and he could not be they if he would. There is naught so high that man will not aspire to it, nothing so low that man cannot stoop to it.

Now come we back to our opening idea, that theories once new and bright and golden, grow old and dull and tarnished, and we chuck them aside, just as we must abandon the old time idea that work is the antipodes of play—that work is the absence of all play, and play the absence of all work ; and therefore we substitute that other and more acceptable theory that work, real, genuine, soulfull work is play, systematized and made profitable as well as pleasurable. That all effort which carries not its own joy with it, apart from compensations and ulterior ends, is drudgery, slavery, serfdom, thralldom, we are prepared to claim. Work deserves a higher type of definition than our lexicographers assign to it. It has even fallen below their definition, and is applied to that which is purely physical effort, tainted with an idea of repugnance.

Thus go our old theories by the board as we ascend the plane of investigation, and bring metaphysics to bear upon our daily duties. Work burdensome ? No. Play profitless ? No, for work is play when rightly adapted to the worker, and play that is profitless, as much play is, is no better play than the play that gets bread at the same time that it conveys happiness to the player. That our happiest moments—moments that fairly thrill the soul—are our working moments, he will aver who is one of those most blest of men who has, after long search or by happy chance struck his affinity of work which he so proudly and earnestly engages in as to make even the player suspicious of his play, while he looks upon the worker as needful of no holiday, since the nice adaptation of his nature to his work makes every day a holiday, and life but one long play-spell.

